# Rare ligamentum flavum cyst causing incapacitating lumbar spinal stenosis: Experience with 3 Chinese patients

**DOI:** 10.1186/1749-799X-5-81

**Published:** 2010-11-04

**Authors:** Alexander Pak-Hin Chan, Tsz Cheung Wong, Koon-Man Sieh, Simon Siu-Man Leung, Kai-Yin Cheung, Kwai-Yau Fung

**Affiliations:** 1 Department of Orthopaedics and Traumatology, Alice Ho Miu Ling Nethersole Hospital, Tai Po, New Territories, Hong Kong SAR, China

## Abstract

Three Chinese patients suffered from severe lumbar spinal stenosis with debilitating symptoms due to a rare condition of ligamentum flavum cysts in the midline of the lumbar spine. This disease is distinct from synovial cyst of the facet joints or ganglion cysts, both intraoperatively and histopathologically. Magnetic Resonance imaging features of the ligamentum flavum cyst are also demonstrated. We share our surgical experiences of identification of the ligamentum flavum cysts, decompression and excision for two of the patients with demonstrably good recovery. This disease should be considered in the differential diagnosis of an extradural instraspinal mass in patients with lumbar spinal stenosis.

## Background

Lumbar spinal stenosis is commonly caused by degenerative conditions, such as herniated nucleus propulsus, or hypertrophy of facet joint or ligamentum hypertrophy. Less common aetiologies include intraspinal extradural masses, including synovial cysts [1], ganglion cysts [2], pseudocysts, haematoma, metastatic tumour. Cyst originated from ligamentum flavum is even more uncommon. Few reported cases of spinal stenosis and radiculopathy were caused by ligamentum flavum cysts and none of these are from the Chinese population [3-8]. We describe our experiences of Chinese patients suffering from spinal stenosis due to ligamentum flavum cysts.

## Case Presentation

### Case 1

A 74-year-old man presented with 1-year history of progressive bilateral leg weakness with left side being more affected. He walked with frame in the most recent 3 months. There was no history of trauma or other constitutional symptoms.

Clinical examination revealed moderate weakness (grade 3/5) of the left ankle dorsiflexion, flexion and extension of the left great toe. Otherwise, motor examination of the other muscle groups was normal. Sensation of both lower limbs was intact. The left knee reflex was diminished whereas both side ankle reflexes were absent.

**Figure 1:**
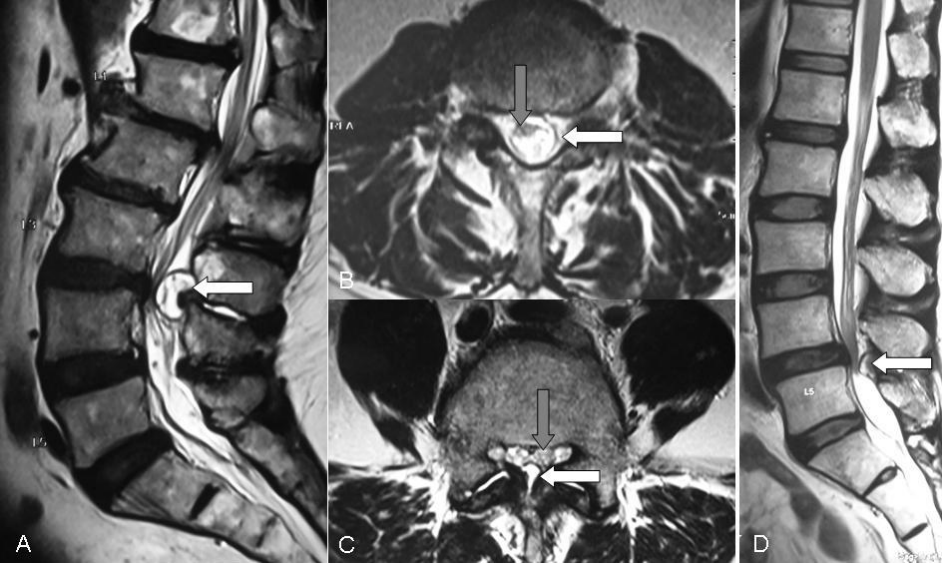
**Patient 1: (A) Sagittal and (B) transverse T2-weighted MRI with contrast of the lumbosacral spine shows a rim enhancing cystic lesion (white arrow) centrally located at the L3/4 level compressing onto the cauda equina (grey arrow)**. Patient 2: (C) Transverse and (D) sagittal T2-weighted MRI image shows Grade I spondylolisthesis and spinal stenosis at the L4/5 level being compressed by a huge cystic structure (white arrow) with a thick cystic wall onto the cauda equina (grey arrow).

Plain radiographs of the lumbosacral spine showed degenerative changes. Blood parameters showed normal white cell count (WCC), erythrocyte sedimentation rate (ESR) and C-reactive protein (CRP). Magnetic Resonance Imaging (MRI) scan showed a cystic lesion, T1 hypointense and T2 hyperintense with moderate rim enhancement in between dural sac and ligamentum flavum at L3/4 level, compressing and displacing cauda equina (Figure 1A, B).

The patient was treated conservatively with a short course of analgesics and physiotherapy. This regimen failed to alleviate the patient's symptoms. Posterior decompression by L3 laminectomy was performed. Intraoperatively, a cyst was noted in the epidural space spanning the whole of ligamentum flavum in a transverse and cranio-caudal direction at L3/4 level. Two separate ganglion cysts were also found on the dorsal side of both L3/4 facet joints. The dorsal side of the ligamentum flavum cyst extended in a space in the L3/4 interspinous space, whereas the ventral wall was densely adhered to the dura. The cauda equina was decompressed by excising the dorsal cyst wall and drainage of clear fluid inside. The ventral wall could not be separated from the dura and was left in-situ. Further decompression of bilateral lateral canals was performed by undercutting of the facet joints. Both descending L4 nerve roots were decompressed from the shoulders to the entry zone of neuroforamina. Histological examination of the resected specimen demonstrated fibrous connective tissue devoid of lining epithelium.

Postoperatively, the patient showed relief from the spinal claudication symptoms and improvements in ankle dorsiflexion, left great toe flexion and extension power to grade 4/5. He could walk with stick.

### Case 2

The second patient was a 56-year-old male optician, who sought orthopaedic consultation because of rapidly progressing bilateral lower limb numbness and weakness for 2 months. Walking tolerance was limited to less than 5 minutes drastically in this period. Left side symptoms were more severe than right side.

Physical examination showed tenderness at lower lumbar spine, weakness of grade 4/5 over left extensor hallucis longus muscle, with decrease in light touch sensation over left L5 dermatome. Straight leg raising test was noted positive at 75 degrees on the left side with tension sign. Lower limb reflexes were intact. All the features were compatible with left sciatica with left L5 radiculopathy.

Radiograph of the lumbosacral spine demonstrated grade I spondylolisthesis at L4/5 level with decreased L4/5 intervertebral disc height. MRI of the same region showed L4/5 disc bulging with severe spinal stenosis (Figure 1C, D).

**Figure 2:**
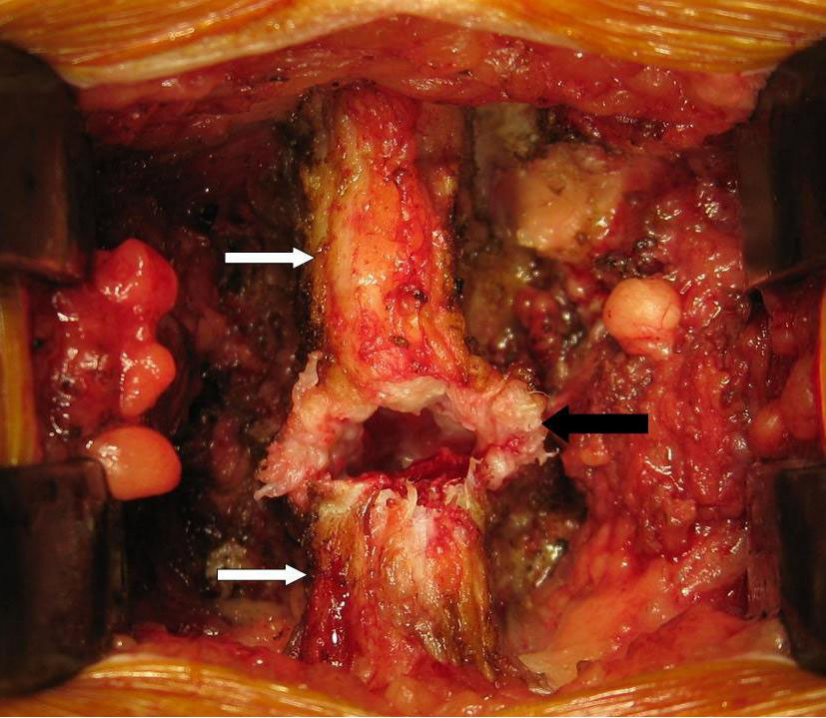
**Intraoperative photo shows the thickened ligamentum flavum cyst wall (black arrow) in between spinous processes (white arrows) upon posterior decompression by laminectomy at L4/5 level**.

Posterior decompression by L4/5 laminectomy was performed. A midline cyst arising from the ligamentum flavum with dense epidural adhesion at the midline was noted (Figure 2). The cyst was freed from the dura and excised with some adherent remnants at the midline. Both descending L5 nerve roots were severely compressed in the lateral canals. L4 and L5 pedicle screw fixation, together with Transforaminal Lumbar Interbody Fusion (TLIF) of the L4/5 levels, were also done to correct the spondylolisthesis. On histological examination, there was dense fibrous tissue identified from the cyst wall.

After the operation, the patient could walk with stick without any claudication symptoms. The left L5 radiculopathy was completely resolved.

### Case 3

The third patient was an 85-year-old lady with known low back pain on and off for years. She also had history of hypertension, hyperlipidaemia, old cerebrovascular accident. She presented with sudden deterioration in back pain with bilateral lower limb numbness for 1-month. Physical examination showed local tenderness at the lower lumbar region. Lower limb neurology was intact. X-ray of the lumbosacral spine showed degenerative scoliosis. MRI of the lumbosacral spine showed postero-central disc protrusion at L3/4, L4/5 and L5/S1, with lumbar spinal stenosis, L4/5 and L5/S1 facet joint hypertrophy. There was a hyperintense cystic lesion at L3/4 region on T2-weighted image over posterolateral aspect of the dura on the left side, compatible with a ligamentum flavum cyst. However, the patient refused decompression operation due to medical comorbidities. She was provided with conservative management with static progress.

## Discussion

In the past, the occurrence of cysts arising from the posterior elements of the spinal canal is termed juxta-facet cysts [9]. However, with more cases and experiences accumulated, there are specific features that we can differentiate the various origins of the cysts. Identifying and differentiating the origin of the cystic lesions have important implications to our surgical planning and the extent of surgical exploration to achieve complete cyst resection from its insertion and minimize the chance of recurrence. Synovial or ganglion cysts of the facet joints have been reported to cause nerve root compression in the lumbar spine [1,2]. Synovial cysts communicate with the facet joint, have a synovial lining (pseudostratified columnar epithelium), and contain clear or xanthochromic fluid [1]. The spinal ganglion cysts do not communicate with the facet joint cavity, have a fibrous tissue wall, and are filled with a viscous, gelatinous material [9]. However, ligamentum flavum cysts represent a unique entity being embedded in the inner surface of ligamentum flavum with no epithelial lining and no association with spinal facets [6-8].

The pathogenesis of ligamentum flavum cysts remains unknown [6-8]. This condition likely reflects degeneration rather than trauma. From the literature available, hypertrophy of the ligamentum flavum, along with ligamentous degeneration and fibrosis, are frequently present and likely to be sequelae of localized spinal trauma [10]. The ligamentum flavum cyst is regarded to be associated with microtrauma due to increased motion at a particular motion segment or segmental instability and local stress associated with degeneration at the level of occurrence [11,12]. Cyst formation may be part of a spectrum of more advanced ligamentous degeneration, which includes, fibrosis and calcification [5]. Patients with ligamentum flavum cysts are also noted to have co-existence of facet joint degeneration, and incidence of degenerative spondylolisthesis varies between 42 and 65% [13]. This phenomenon was also noted in our first and second patient respectively.

Most of the reported ligamentum flavum cysts in the literature were located laterally within the spinal canal, unlike the central location of the cysts in our first two patients. Improvements in pain and neurological function in the majority of patients suffering from laterally located ligamentum flavum cysts after surgical decompression have been documented [6,8].

Dense adhesions to the dura entail surgical difficulties for complete excision of the ligamentum flavum cysts, which can minimize recurrence. Dural tear might complicate the decompression surgery and cerebrospinal fluid fistula can be one of the most unfavourable surgical sequela.

The natural history of this rare entity is unknown. The progression of the symptoms can vary from acute to subacute, as illustrated in the difference in symptom duration and severity of our three patients. Whether a cyst resorbs over time is unknown. The role of initial non-operative treatment in the setting of clinical symptoms is warranted. Operation is reserved for those with persistent neurological symptoms and/or severe leg pain refractory to non-operative treatment. Decompression of the cysts contents and preferably, subsequent resection of the cyst lining can bring immediate relief of the patient's radicular symptoms.

## Conclusions

The origin of ligamentum flavum cyst is unknown. The occurrence is extremely rare, but it should remain in the differential diagnosis of any extradural intraspinal mass and neurogenic claudication or lumbar radiculopathy. This condition is surgically treatable.

## Competing interests

The authors declare that they have no competing interests.

## Authors' contributions

PHC, KMS and TCW were involved in the operative work-up, rehabilitation and preparation of the manuscript. KYC was the chief surgeon of the operations, while KMS, SML, PHC and TCW assisted the operations. KYF initiated and coordinated the operative planning. All authors contributed and approved the final manuscript.

## Authors' information

Alexander Pak-Hin Chan holds the position of Resident, Department of Orthopaedics and Traumatology, Alice Ho Miu Ling Nethersole Hospital, Hong Kong SAR, China

Tsz-Cheung Wong holds the position of Resident, Department of Orthopaedics and Traumatology, Alice Ho Miu Ling Nethersole Hospital, Hong Kong SAR, China

Koon-Man Sieh holds the position of Specialist, Department of Orthopaedics and Traumatology, Alice Ho Miu Ling Nethersole Hospital, Hong Kong SAR, China

Simon Siu-Man Leung holds the position of Associate Consultant, Department of Orthopaedics and Traumatology, Alice Ho Miu Ling Nethersole Hospital, Hong Kong SAR, China

Kai-Yin Cheung holds the position of Consultant, Department of Orthopaedics and Traumatology, Alice Ho Miu Ling Nethersole Hospital, Hong Kong SAR, China

Kwai-Yau Fung holds the position of Chief-of-service, Department of Orthopaedics and Traumatology, Alice Ho Miu Ling Nethersole Hospital, Hong Kong SAR, China

## Consent

Written informed consent was obtained from the patients of this case report and accompanying images. A copy of the written consent is available for review by the Editor-in-Chief of this journal.
